# Transcriptome‐Wide Association Uncovers LncRNAs Controlling Seed Weight in Soybean

**DOI:** 10.1002/advs.202416794

**Published:** 2025-08-11

**Authors:** Xiang Wang, Xinyu Jiang, Xiaobo Yuan, Zhipeng Zhou, Zhenwei Zhao, Longfei Wang, Wu Jiao, Wenxue Ye, Qingxin Song

**Affiliations:** ^1^ State Key Laboratory of Crop Genetics & Germplasm Enhancement and Utilization Jiangsu Collaborative Innovation Center for Modern Crop Production Nanjing Agricultural University No. 1 Weigang Nanjing Jiangsu 210095 China

**Keywords:** eQTL, lncRNAs, seed weight, soybean, TWAS

## Abstract

Long non‐coding RNAs (lncRNAs) are emerging as key regulators of various biological processes in plants, yet their role in seed weight regulation remains underexplored. To systematically investigate lncRNAs associated with seed weight variation, transcriptome‐wide association studies (TWAS) are performed across a diversity panel of 238 soybean accessions. The analysis identifies 175 and 50 TWAS‐significant lncRNAs at 14 and 21 days after flowering, respectively. Functional validation reveals that two candidate lncRNAs positively regulate seed weight through transcriptional modulation of key genes in seed development pathways. Additionally, an lncRNA‐lncRNA co‐expression module is identified as significantly correlated with seed weight. The eQTL analysis reveals dynamic and static eQTLs that coordinately modulate lncRNA expression across seed developmental stages. Notably, integration of major‐effect proximal and minor‐effect distal eQTLs enhances phenotype prediction of seed weight, underscoring the significant impact of distal eQTLs on phenotypic variation. These findings elucidate the intricate mechanisms by which lncRNAs contribute to seed weight regulation, offering novel insights for enhancing crop yield.

## Graphical abstract

This study employs transcriptome‐wide association studies (TWAS) to identify 201 long non‐coding RNAs (lncRNAs) associated with seed weight. Expression quantitative trait locus (eQTL) analysis reveals dynamic and static regulatory variants controlling lncRNA expression across development. Integrating proximal and distal eQTLs improves phenotype prediction, highlighting the contribution of trans‐regulatory variation. These findings elucidate lncRNA‐mediated mechanisms underlying seed weight.

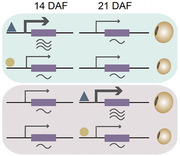

## 1 Introduction

A large and significant portion of the human genome is transcribed into RNA, but less than 2% of RNAs are actually translated into functional protein products.^[^ ^1^ ^]^ The residual transcripts are normally classified as non‐coding RNAs (ncRNAs). Of these ncRNAs, long non‐coding RNAs (lncRNAs) are a group of transcripts longer than 200 nucleotides with low/no protein‐coding potential.^[^ ^2^ ^]^ In the past decade, thousands of lncRNAs have been identified in a wide range of plant species, including *Arabidopsis*, rice, and soybean.^[^ ^3^ ^]^ Emerging evidence suggests that lncRNAs act through diverse mechanisms, including chromatin remodeling, transcriptional interference, and miRNA sponging, to fine‐tune seed size and yield‐related traits.^[^ ^4^ ^]^ The lncRNA *MIS‐SHAPEN ENDOSPERM* (*MISSEN*) directly interacts with a helicase family protein to regulate endosperm patterning in rice.^[^ ^5^ ^]^ Ectopic expression of *MISSEN* perturbs endosperm development, leading to aberrant seed morphology and compromised grain filling.

Soybean (*Glycine max*) is the most widely cultivated legume due to its high protein content, oil production, and nitrogen‐fixing ability.^[^ ^6^ ^]^ Cultivated soybean was domesticated from its wild progenitor *Glycine soja* in China 6000–9000 years ago.^[^ ^6^ ^]^ Artificial selection led to notable changes in both morphological and physiological characteristics, such as enlarged seed size and decreased seed dormancy.^[^ ^7^ ^]^ Seed weight represents a crucial agronomic trait that plays a direct role in shaping both crop yield and quality. To date, over 300 quantitative trait loci (QTLs) implicated in the regulation of seed size and weight have been identified in soybean via linkage analysis and genome‐wide association studies (GWAS) (SoyBase, https://soybase.org/). A number of genes that control seed weight have been isolated and functionally characterized in soybean,^[^ ^8^ ^]^ such as *Phosphatase 2C‐1* (*GmPP2C‐1*), *BIG SEEDS1* (*GmBS1*), and *KINASE‐INDUCIBLE DOMAIN INTERACTING 8* (*GmKIX8‐1*). However, the association of lncRNAs with seed weight has been rarely reported in soybean, and the underlying molecular mechanisms governing how genetic variation impacts lncRNA expression remain ambiguous.

To identify seed weight‐related lncRNAs and elucidate the mechanisms by which genetic variations modulate their expression during seed development, we employed population re‐sequencing and transcriptomic data, in conjunction with transcriptome‐wide association studies (TWAS), expression quantitative trait locus (eQTL) analysis, and lncRNA co‐expression module assessment. Subsequent genetic transformation assays validated the positive regulatory role of two lncRNAs in enhancing seed weight in soybean. These findings not only shed light on the biological roles of lncRNAs but also elucidate their intricate regulatory mechanisms.

## 2 Results

### 2.1 Global Expression Patterns of lncRNAs in Soybean

**Figure 1:**
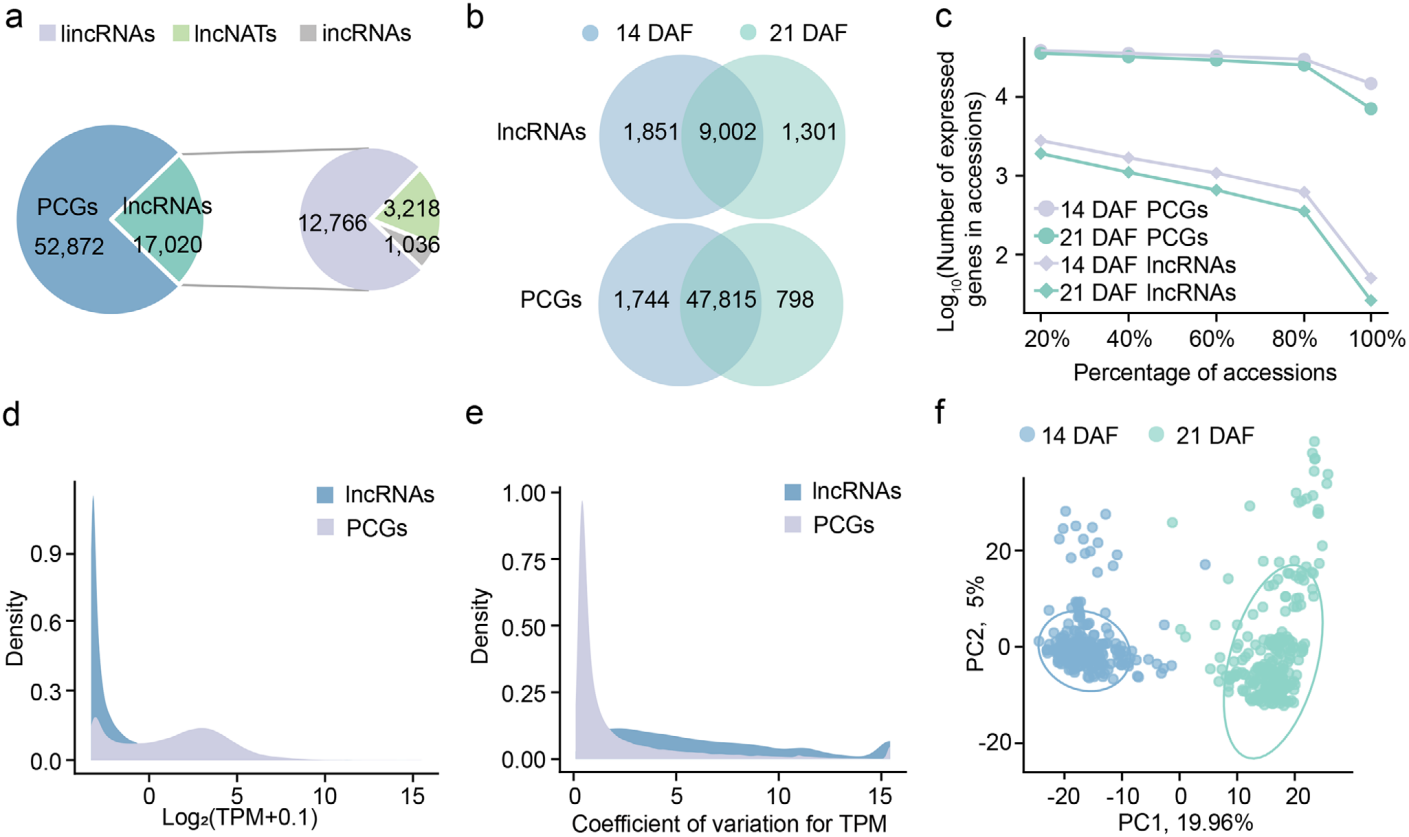
Expression variation of lncRNAs among 238 soybean accessions. a) The number of annotated PCGs and identified lncRNAs in the soybean genome. b) The number of PCGs and lncRNAs expressed at two stages of seed development. Expressed PCGs and lncRNAs were defined by TPM > 0 in at least one accession. c) The number of PCGs or lncRNAs expressed in different percentages of soybean accessions at 14 DAF and 21 DAF. d,e) The density distribution of expression levels (d) and coefficient of variation values (e) for PCGs and lncRNAs across 476 accessions. f) PCA plot of the first two principal components of 476 RNA‐seq samples.

To dissect global features and potential functions of lncRNAs in soybean, we systematically identified lncRNAs across the entire genome using 770 published strand‐specific transcriptome datasets from various tissues at different developmental stages in the NCBI and CNCB databases (Table S1, Supporting Information). Following reference‐guided transcriptome assembly, we identified 17020 putative lncRNAs (> 200 nt) by excluding potential protein‐coding transcripts through a series of stringent filtration steps, as well as loci generating transcripts exhibiting sequence similarity to ribosomal RNAs (rRNAs), transfer RNAs (tRNAs), and small RNAs (Figure S1a, Supporting Information). These lncRNAs were classified into three categories: transcripts originating from intergenic regions (lincRNAs, 12766), transcripts from the antisense strand of protein‐coding genes (PCGs) (lncNATs, 3218), and transcripts from intronic regions of PCGs (incRNAs, 1036) (**Figure** **1**a). To investigate the expression variations of lncRNAs in soybean, we generated expression patterns of seeds at 14 days after flowering (DAF) for 238 widely collected soybean accessions using 3′ RNA‐seq (Figure S1b and Table S2, Supporting Information), in conjunction with transcriptome datasets of seeds at 21 DAF in our previous study.^[^ ^9^ ^]^ Soybean seeds reached half of the pod cavity volume at 14 DAF and achieved full cavity filling by 21 DAF, marking two pivotal phases of rapid seed expansion that ultimately determine final grain size. On average, we obtained 2.8 million sequencing reads for each accession (Table S2, Supporting Information).

In the soybean population, a total of 10853 and 10303 lncRNAs were found to be expressed in seeds at 14 and 21 DAF, respectively (Figure 1b), and these lncRNAs were selected for subsequent analysis. Furthermore, we detected 49559 and 48613 expressed PCGs in seeds at 14 and 21 DAF, respectively (Figure 1b). About 90% (47815) of PCGs demonstrated expression in seeds at both 14 and 21 DAFs. In contrast, a modest fraction (53%, 9002) of lncRNAs were detected in both developmental stages, indicating spatiotemporal‐specific expression patterns of lncRNAs. Notably, only 0.16–0.29% (50/17020, 14 DAF; 28/17020, 21 DAF) of lncRNAs exhibited expression across all 238 accessions, whereas ≈13–28% (14776/52872, 14 DAF; 7082/52872, 21 DAF) of PCGs were universally expressed in all accessions (Figure 1c; Table S3, Supporting Information). Many (47%, 14 DAF; 49%, 21 DAF) lncRNAs were expressed in fewer than 20% of soybean accessions (Figure 1c). Furthermore, lncRNAs exhibited lower expression levels and larger coefficients of expression variations compared to PCGs (Figure 1d,e). These results indicate limited conservation of lncRNAs among soybean accessions. A principal component analysis (PCA) based on lncRNA expression levels demonstrated a pronounced differentiation between seeds at two developmental stages (Figure 1f).

### 2.2 Identification of lncRNAs Associated with Seed Weight by TWAS

**Figure 2:**
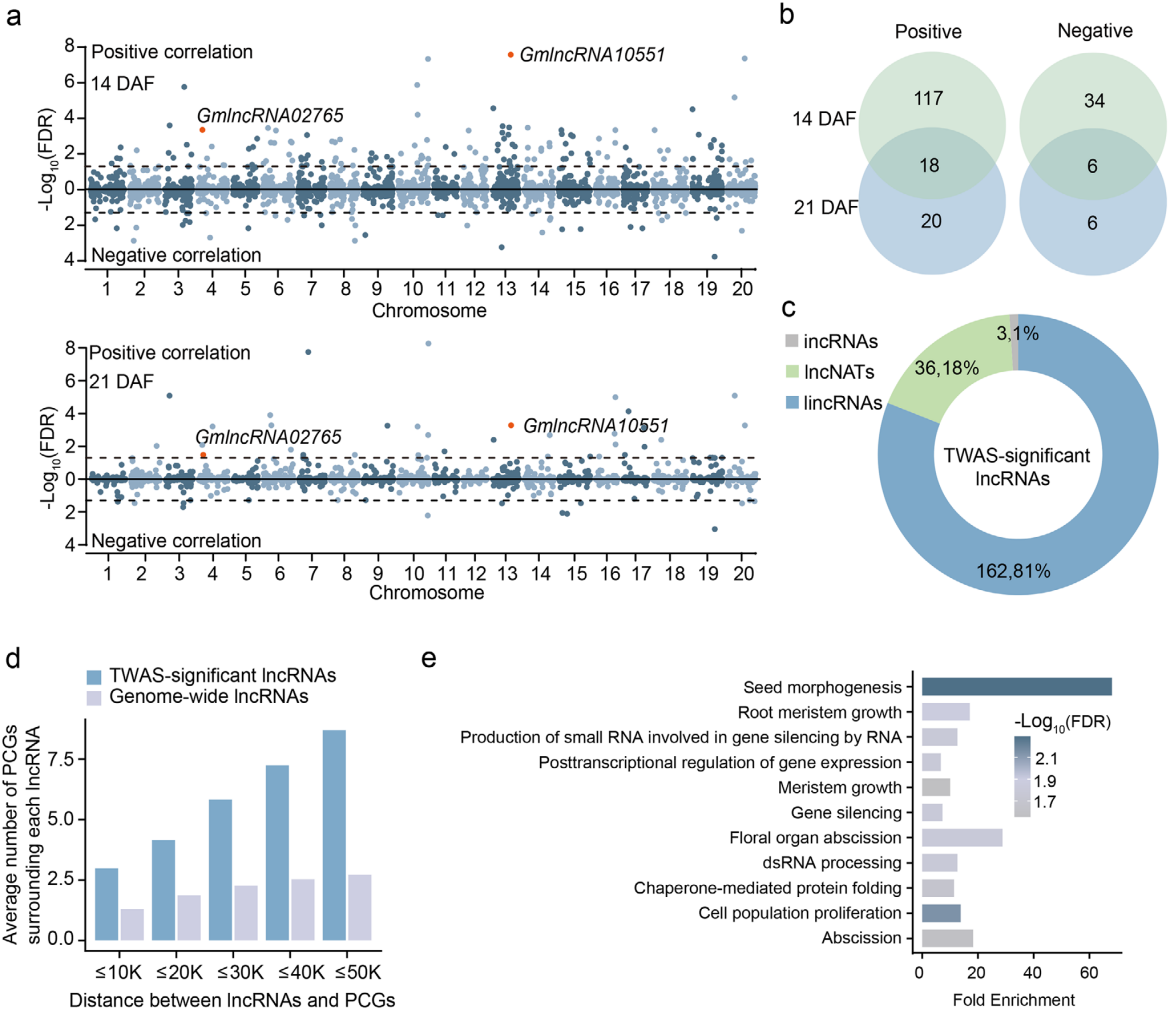
TWAS for seed weight based on lncRNA expression at 14 DAF and 21 DAF. a) Manhattan plots displaying the correlation between lncRNAs expression and 100‐seed weight (top panel, 14 DAF; bottom panel, 21 DAF). Each dot represents the FDR value of the Pearson correlation between lncRNAs expression and 100‐seed weight. The horizontal dashed lines indicate the significance threshold for correlation (FDR < 0.05). b) Overlapped number of TWAS‐significant lncRNAs at 14 DAF and 21 DAF. c) The distribution of TWAS‐significant lncRNAs. d) Average number of PCGs surrounding lncRNAs. e) GO enrichment analysis for *cis*‐regulated target PCGs of TWAS‐significant lncRNAs.

TWAS has been employed to elucidate the pivotal genes that regulate crucial agronomic traits in crops.^[^ ^9^, ^10^ ^]^ In order to establish a connection between lncRNAs and seed weight, TWAS was performed using expression levels of lncRNAs and 100‐seed weight phenotypes of 238 cultivated soybean (**Figure** **2**a). Totally, there were 175 and 50 lncRNAs significantly associated with seed weight at 14 DAF and 21 DAF, respectively (FDR < 0.05) (Figure 2a; Table S4, Supporting Information), of which 24 significant lncRNAs were detected at both seed development stages (Figure 2b). The Pearson correlation coefficients between the expression levels of lncRNAs and seed weight demonstrated a positive correlation at two developmental stages (Figure S2, Supporting Information). Most TWAS‐significant lncRNAs (81%) were derived from intergenic regions (Figure 2c). Notably, PCGs were significantly enriched in 10–50 kb surrounding regions of TWAS‐significant lncRNAs relative to genome‐wide lncRNAs (Figure 2d). LncRNAs could exert regulatory influences on target genes, operating in either *cis* or *trans* fashion.^[^ ^2^ ^]^ *Cis*‐acting lncRNAs regulate nearby genes through local chromatin or expression modulation, while *trans*‐acting lncRNAs influence distant targets via protein/RNA interactions. To elucidate the potential mechanisms governing seed weight regulation by lncRNAs, we predicted 149 *cis*‐regulated PCGs for TWAS‐significant lncRNAs following a previously reported methodology (Table S5, Supporting Information).^[^ ^11^ ^]^ Notably, these targets included key seed weight regulators, including *GA20OX*, *ARF2*, *ABA2*, and *DA1* (Table S5, Supporting Information).^[^ ^12^ ^]^ Gene Ontology (GO) enrichment analysis revealed that 11 GO functional categories were significantly overrepresented among the target genes, such as seed morphogenesis and cell population proliferation (Figure 2e).

### 2.3 eQTL Mapping for lncRNAs

**Figure 3:**
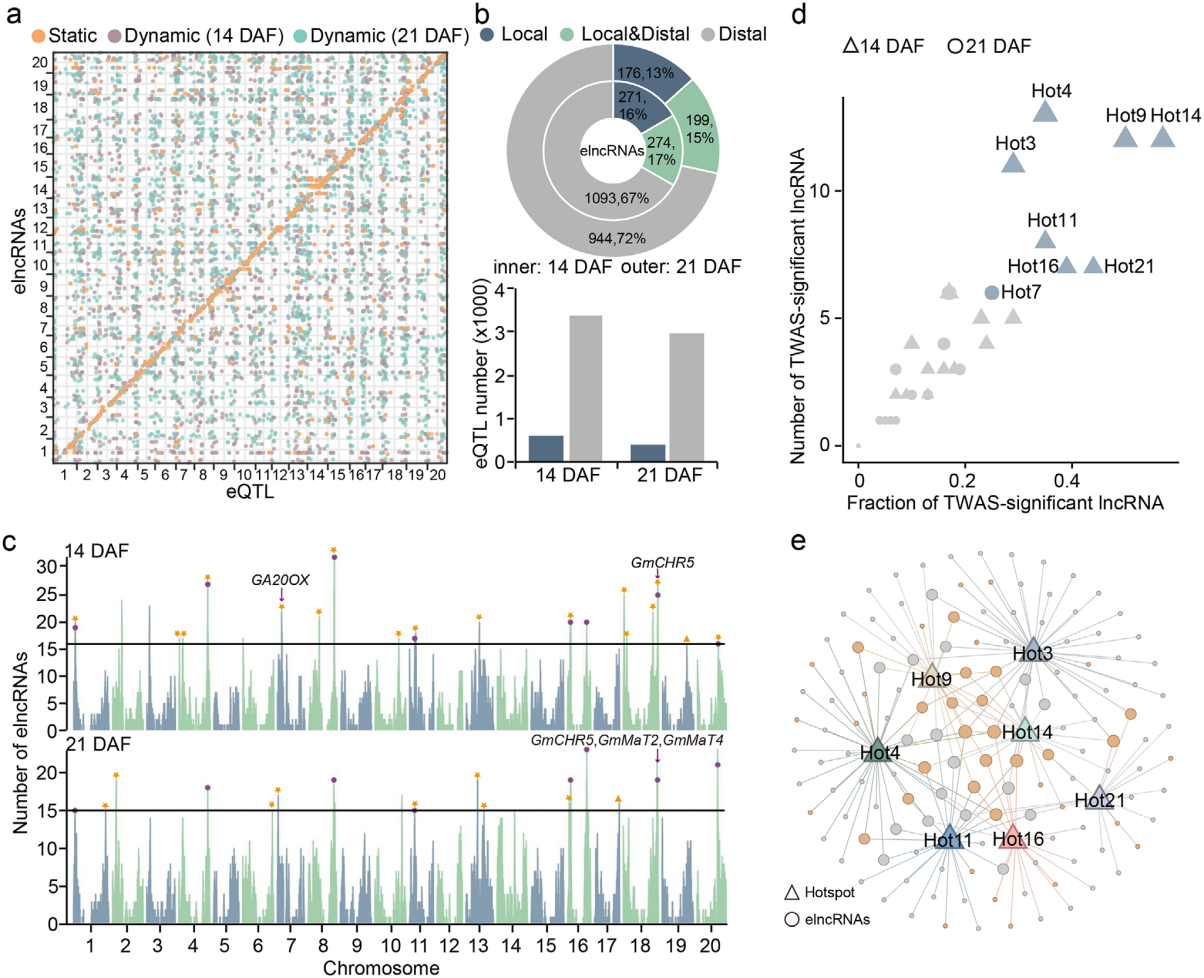
Identification of eQTLs and hotspots for lncRNAs. a) The dot plot represents the associations of eQTLs and their mapped elncRNAs in the genome. b) The percentage of elncRNAs regulated by different types of eQTLs (top) and the number of local and distal eQTLs (bottom) at 14 DAF and 21 DAF. c) Identification of eQTL hotspots at 14 DAF (top) and 21 DAF (bottom). Solid lines represent the threshold number of lncRNAs for eQTL hotspots (14 DAF, 16; 21 DAF, 15). The purple dots represent the overlapped eQTL hotspots at both 14 DAF and 21 DAF. The orange asterisks and triangles represent eQTL hotspots that overlap with known QTLs and genes associated with seed traits, respectively. d) Identification of eQTL hotspots (blue symbole) whose target elncRNAs are significantly enriched in TWAS‐significant lncRNAs (hypergeometric test, *P* < 0.01). e) Genetic network formed by eQTL hotspots and mapped elncRNAs. The size of the dots represents the in‐degree of hotspot‐elncRNA connectivity.

In order to elucidate the effects of genetic variants on lncRNA expression at two seed development stages, we conducted eQTLs mapping of lncRNA (elncRNA) at 14 and 21 DAF using SNP datasets and lncRNA expression datasets from 238 soybean accessions (**Figure** **3**a). We identified 3948 and 3361 eQTLs for 1638 and 1319 elncRNAs at 14 and 21 DAF, respectively (Figure 3b). Based on the distance (threshold of 1 Mb) between eQTLs and elncRNAs, we uncovered 591 local and 3357 distal eQTLs for 545 and 1367 elncRNAs at 14 DAF, respectively. At 21 DAF, 402 local and 2959 distal eQTLs were detected for 375 and 1143 elncRNAs, respectively (Figure 3b). Additionally, 17% and 15% of elncRNAs were regulated by both local and distal eQTLs at 14 DAF and 21 DAF, respectively (Figure 3b).

Although the majority of elncRNAs were regulated by only one eQTL, we detected 22 and 20 distal eQTL hotspots, which could regulate multiple (> 15) elncRNAs at 14 DAF and 21 DAF, respectively (Figure 3c; Figure S3a, Supporting Information), of which 8 eQTL hotspots overlapped between the two developmental stages (Figure 3c). Notably, most eQTL hotspots overlapped with previously reported QTLs and known genes related to seed traits (Figure 3c), such as *GA20OX* for seed weight, *GmCHR5*, *GmMaT2*, and *GmMaT4* for isoflavone content.^[^ ^12^, ^13^ ^]^ These findings indicate that the identified eQTL hotspots likely contribute to seed trait variation through direct or indirect modulation of lncRNA expression. The TWAS‐significant lncRNAs were significantly enriched in target elncRNAs associated with 7 eQTL hotspots at 14 DAF and 1 eQTL hotspot at 21 DAF (Figure 3d), indicating the important role of eQTL hotspots in regulating seed development. Interestingly, the elncRNAs regulated by these eQTL hotspots at 14 DAF could form a genetic network through eQTL‐elncRNA connection (Figure 3e). This finding suggests that eQTL hotspots may function cooperatively through common downstream elncRNAs to regulate complex biological processes, shedding light on the complex regulatory relationships between genetic variants and lncRNAs during seed development. Based on published resequencing data for wild and cultivated soybeans, including landraces and cultivars, we found that 7 eQTL hotspots at 14 DAF and 5 eQTL hotspots at 21 DAF experienced strong artificial selection during the domestication of soybean (Figure S3b, Supporting Information).

### 2.4 Dynamic Regulation of lncRNA Expression

**Figure 4:**
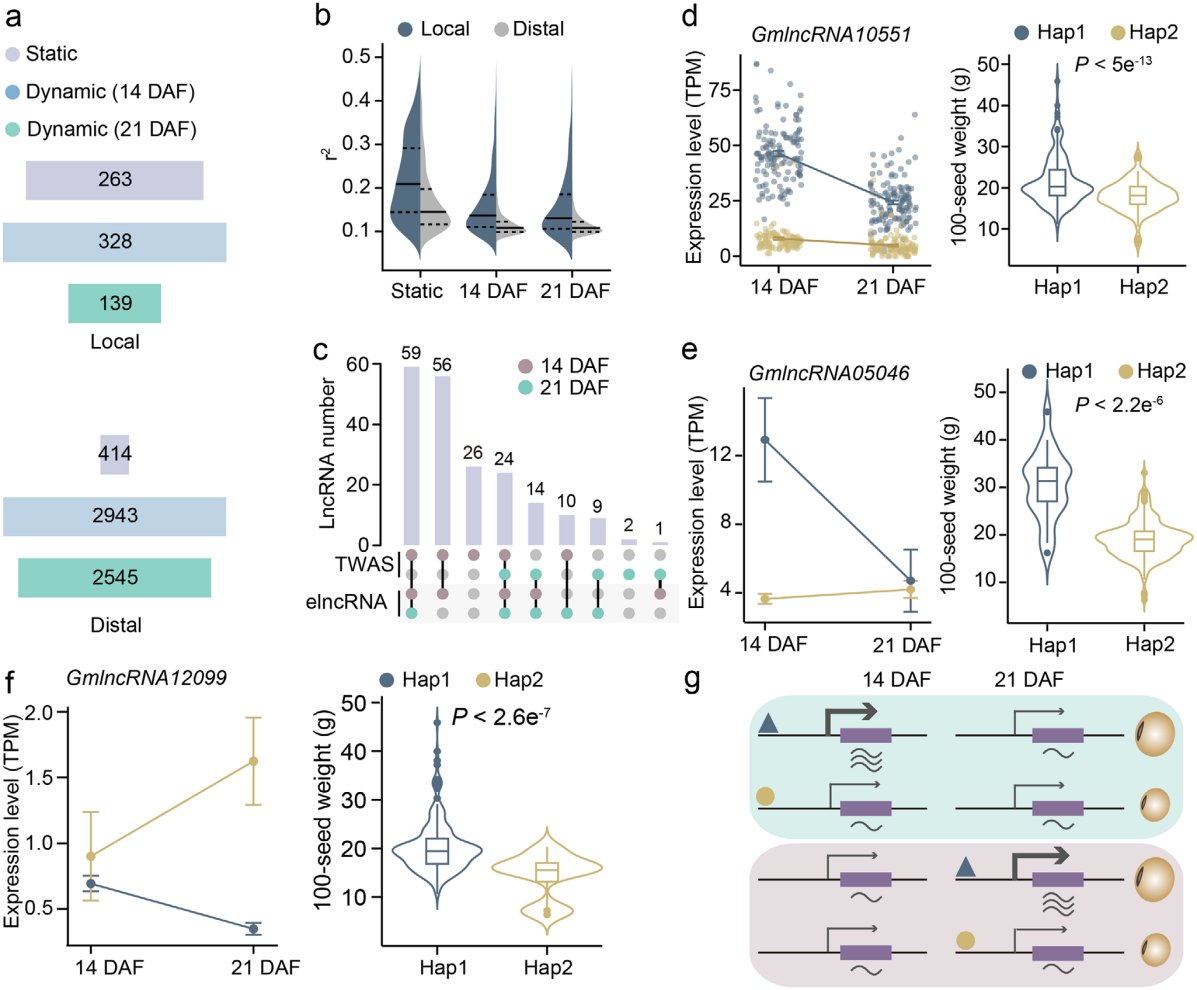
Identification of static and dynamic eQTLs of lncRNA. a) The number of dynamic and static eQTLs. b) Distribution of explained variance (*r* ^2^) of static and dynamic eQTLs for their corresponding elncRNAs. The dashed lines indicate the 0.25 and 0.75 quantiles. c) UpSet plot showing the overlap among TWAS‐significant lncRNAs and elncRNAs at 14 DAF and 21 DAF. d–f) Examples of TWAS‐significant lncRNAs regulated by static and dynamic eQTLs. The distributions of expression levels (left) and 100‐seed weight (right) are shown for two haplotypes of eQTLs associated with TWAS‐significant lncRNAs: *GmlncRNA10551* regulated by static eQTL (d), *GmlncRNA05046* regulated by dynamic eQTL at 14 DAF (e), and *GmlncRNA12099* regulated by dynamic eQTL at 21 DAF (f). g) A schematic of dynamic eQTLs regulating TWAS‐significant lncRNAs to control seed weight. Dynamic eQTLs may influence the expression levels of TWAS‐significant lncRNAs at specific developmental stages, thereby affecting seed weight.

The lncRNAs exhibited dramatic expression divergence across different seed developmental stages (Figure 1f), which suggests dynamic expression data may unveil additional dimensions in eQTL analysis, enabling the identification of genetic variants with transient effects.^[^ ^10^, ^14^ ^]^ The eQTLs detected at both stages were defined as static eQTLs, while those detected only at 14 or 21 DAF were defined as dynamic eQTLs (**Figure** **4**a). We identified 263 static local eQTLs, 328, and 139 dynamic local eQTLs at 14 DAF and 21 DAF, respectively. For distal eQTLs, we detected 414 static eQTLs, 2943 dynamic eQTLs at 14 DAF, and 2545 dynamic eQTLs at 21 DAF. As expected, static eQTLs exhibited a larger effect on lncRNA expression compared to dynamic eQTLs, and their associated lncRNAs maintained more stable expression levels across both stages relative to lncRNAs targeted by dynamic eQTLs (Figure 4b; Figure S4a, Supporting Information). Among the 201 TWAS‐significant lncRNAs, 173 were mapped by eQTLs; of these, 24 were regulated by static eQTLs, while the remaining were governed by dynamic eQTLs (Figure 4c). The static eQTLs could mediate the expression divergence of lncRNAs among accessions in both developmental stages. For example, the TWAS‐significant lncRNA *GmlncRNA10551* was mapped by a static eQTL encompassing two haplotypes (Hap1 and Hap2) (Figure 4d; Figure S4d, Supporting Information). Soybean accessions harboring Hap1 exhibited significantly elevated expression levels of *GmlncRNA10551* at both developmental stages, as well as increased 100‐seed weight, compared to those carrying Hap2 (Figure 4d). In contrast to static eQTLs, dynamic eQTLs may mediate the expression divergence of lncRNAs among accessions in specific developmental stages. For instance, the TWAS‐significant lncRNA *GmlncRNA05046* that was associated with a dynamic eQTL at 14 DAF demonstrated a positive correlation between its expression levels and 100‐seed weight (Figure S4b, Supporting Information). Soybean accessions with the superior haplotype (Hap1) exhibited higher expression levels of *GmlncRNA05046* at 14 DAF but not at 21 DAF and increased 100‐seed weight than those with inferior haplotype (Hap2) (Figure 4e; Figure S4d, Supporting Information). Similarly, *GmlncRNA12099*, mapped by a dynamic eQTL at 21 DAF, demonstrated a negative correlation between its expression levels and 100‐seed weight (Figure S4c, Supporting Information). The soybean accessions harboring the superior haplotype (Hap1) for heavier seed weights exhibited diminished expression levels at 21 DAF but not at 14 DAF compared to those with inferior haplotype (Hap2) (Figure 4f; Figure S4d, Supporting Information). These findings suggest that both dynamic and static eQTLs can modulate lncRNA expression at specific or multiple developmental stages, thereby influencing seed weight (Figure 4g).

### 2.5 Integration of Local and Distal eQTLs Enhances Phenotype Prediction

**Figure 5:**
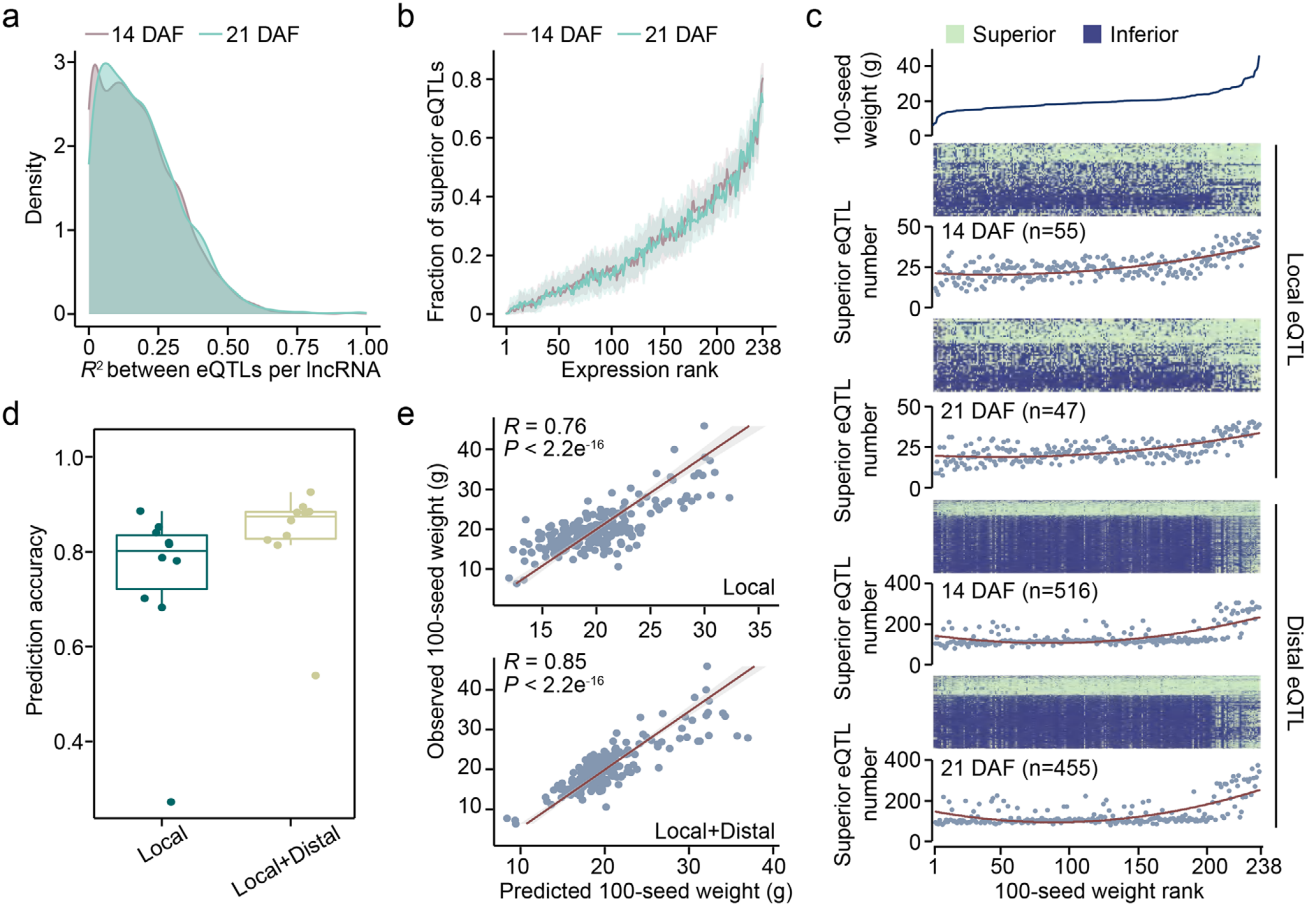
The aggregation of superior eQTLs associated with TWAS‐significant lncRNAs in soybean accessions. a) Density plot for the pairwise *R* ^2^ (LD) between eQTLs for each elncRNA. b) Average fraction of aggregated superior eQTLs for each rank of lncRNA expression level. c) The distribution of superior local or distal eQTLs in 238 accessions. The line chart illustrates the 100‐seed weight in each accession. The heatmap shows the distribution of superior and inferior eQTLs. The scatter plot depicts the superior eQTL counts in each accession. The *x*‐axis represents the 238 accessions ranked by 100‐seed weight. d,e) The prediction accuracy for 100 seed‐weight using different combinations of local and distal eQTLs for TWAS‐significant lncRNAs. Prediction accuracy is quantified by the correlation coefficient (*R*).

Although most elncRNAs were regulated by a single eQTL (Figure S3a, Supporting Information), the majority (73%, 126/173) of TWAS‐significant elncRNAs were associated with multiple (≥ 2) eQTLs. We calculated the pairwise linkage disequilibrium (*R* ^2^) among eQTLs for each TWAS‐significant elncRNA and observed that these eQTLs were independent of each other, with an average *R* ^2^ of 0.2 (**Figure** **5**a). We hypothesized that the aggregation of genetic regulatory loci for TWAS‐significant lncRNAs was essential to attain elevated expression levels of these lncRNAs. To test it, we defined the haplotype of eQTLs with a positive effect on lncRNA expression as a superior haplotype. For each TWAS‐significant lncRNA, we ranked soybean accessions based on lncRNA expression levels and documented the proportion of eQTLs with superior haplotypes among the total eQTLs identified. On average, soybean accessions with higher expression levels of lncRNAs aggregated a larger number of superior eQTLs (Figure 5b), indicating the effects of eQTLs on lncRNA expression are not redundant.

To examine whether aggregation of local and distal eQTLs of TWAS‐significant lncRNAs could enhance seed weight, we ranked 238 accessions by their 100‐seed weight values and tallied the number of local and distal eQTLs with superior haplotypes that showed positive effects on seed weight (Figure 5c). As seed weight increased, the number of local eQTLs with superior haplotypes also gradually increased (Figure 5c). Notably, even in soybean accessions with smaller seed weight, approximately half of local eQTLs exhibited superior haplotypes (Figure 5c). In contrast to local eQTLs, distal eQTLs with superior haplotypes were mainly concentrated in a small subset of soybean accessions with extremely high seed weight (Figure 5c). To further evaluate the impact of distal eQTLs on phenotype variation, we examined whether their inclusion alongside local eQTLs could improve the prediction accuracy of seed weight. We found that the incorporation of distal eQTLs resulted in a 9% enhancement in predictive accuracy relative to the utilization of local eQTLs alone (Figure 5d,e). These results underscore the significant contribution of distal eQTLs to phenotype variation, highlighting the importance of integrating both major‐effect proximal and minor‐effect trans‐regulatory loci of trait‐associated lncRNAs in breeding to optimize crucial regulatory networks and pathways for complex traits.

### 2.6 Co‐Expression Modules of lncRNAs Involved in Seed Weight Regulation

**Figure 6:**
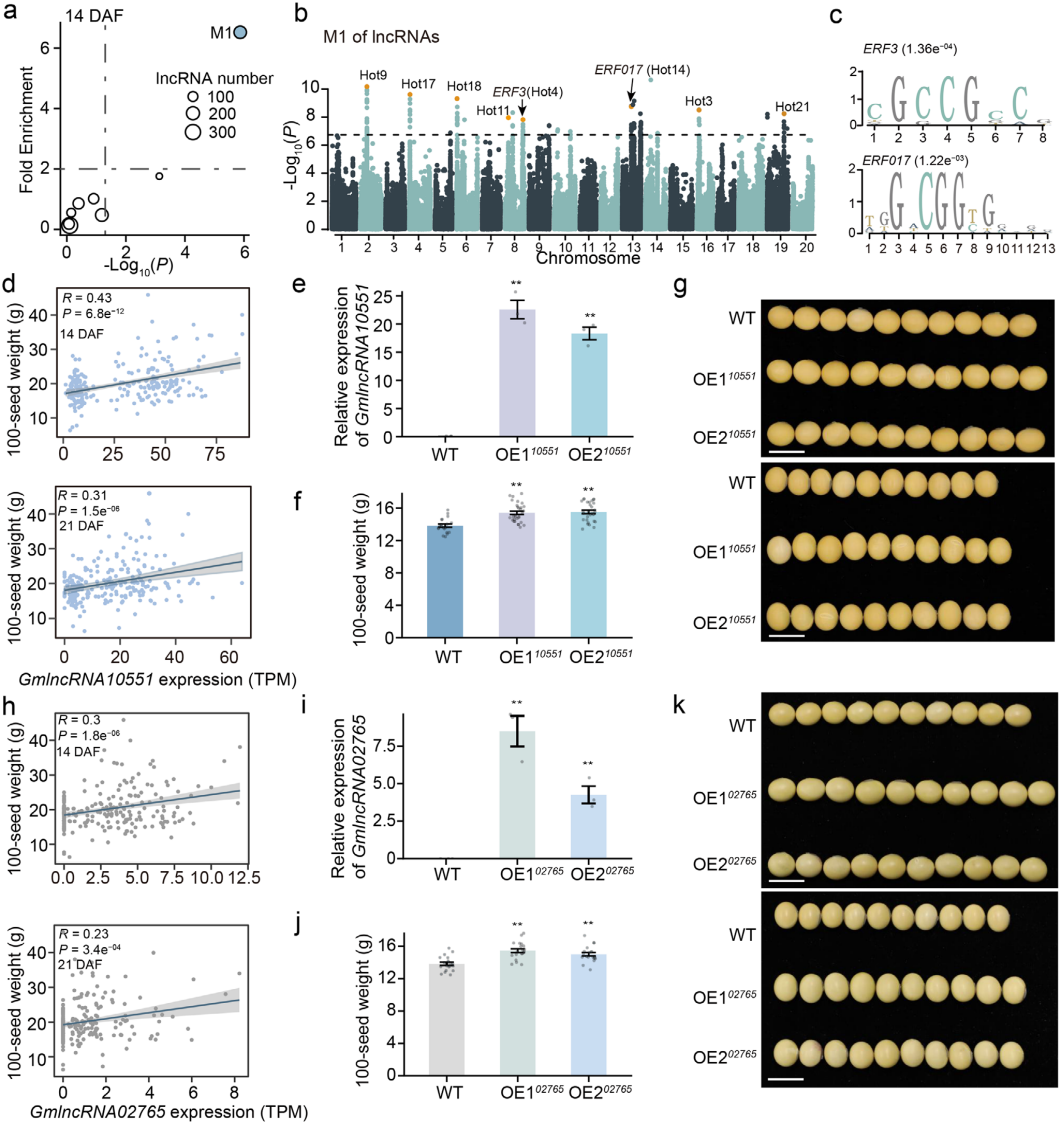
Functional characterization of TWAS‐significant lncRNAs in regulating seed weight. a) Identification of the key co‐expression module of lncRNAs regulating 100‐seed weight at 14 DAF. The *y*‐axis represents the fold enrichment of TWAS‐significant lncRNAs in each co‐expression module. The *x*‐axis represents the *P* value of the Pearson correlation between co‐expression modules and 100‐seed weight. The horizontal and vertical dashed lines represent the corresponding threshold (Fold Enrichment ≥ 2; *P* < 0.05). b) Manhattan plot of GWAS analysis for module M1 at 14 DAF. Dashed lines represent the significance threshold for QTLs regulating module M1 (*P* < 1.85e^−7^). The orange dots represent GWAS loci of M1 that overlap with eQTL hotspots. c) Enrichment of *ERF3* and *ERF017* binding motifs in the promoter sequences of lncRNAs in M1. d) Scatter plot showing the correlation between expression of *GmlncRNA10551* and 100‐seed weight at 14 DAF and 21 DAF. e) Relative expression levels of *GmlncRNA10551* in overexpression lines and WT. The expression levels of lncRNAs were normalized to *GmTublin* using RT‐qPCR (n = 3 biological replicates). Statistical analysis was performed using a two‐tailed *t*‐test. Data are presented as means ± SEM. ^**^ *P* < 0.01. f) Comparison of 100‐seed weight between *GmlncRNA10551* transgenic lines and WT (n = 20, 30, 30 biological independent plants for WT, OE1, and OE2, respectively). Statistical analysis was performed using a two‐tailed *t*‐test. Data are presented as means ± SEM. ^**^ *P* < 0.01. g) Comparison of seed length (top) and seed width (bottom) between *GmlncRNA10551* transgenic lines and WT. Scale bar: 1 cm. h) Scatter plot showing the correlation between expression of *GmlncRNA02765* and 100‐seed weight at 14 DAF and 21 DAF. i) Relative expression levels of *GmlncRNA02765* in overexpression lines and WT. The expression levels of lncRNAs were normalized to *GmTublin* using RT‐qPCR (n = 3 biological replicates). Statistical analysis was performed using a two‐tailed *t*‐test. Data are presented as means ± SEM. ^**^ *P* < 0.01. j) Comparison of 100‐seed weight between *GmlncRNA02765* transgenic lines and WT (n = 20 biological independent plants for WT, OE1 and OE2, respectively). Statistical analysis was performed using a two‐tailed *t*‐test. Data are presented as means ± SEM. ^**^ *P* < 0.01. k) Comparison of seed length (top) and seed width (bottom) between *GmlncRNA02765* overexpression transgenic lines and WT. Scale bar: 1 cm.

To provide a view of seed weight variations arising from coordinated lncRNA regulation, we inferred lncRNA‐lncRNA co‐expression networks based on the expression correlation between lncRNAs. Of the 8 co‐expression lncRNA modules constructed, 2 modules (M1 and M2) were significantly correlated with 100‐seed weight at 14 DAF (**Figure** **6**a). Meanwhile, one module of 15 lncRNA modules exhibited significant correlation with 100‐seed weight at 21 DAF (Figure S5, Supporting Information). Notably, we found that TWAS‐significant lncRNAs were over‐represented in module M1 but not in other modules (Figure 6a; Table S12, Supporting Information). As module M1 not only showed significant correlation with 100‐seed weight but also contained the highest fraction of TWAS‐significant lncRNAs, we focused on the genetic mechanism underlying the regulation of module M1 in subsequent analysis.

To detect genomic loci tending to distally regulate the module M1, we performed GWAS using the expression pattern of module M1 as a phenotype. We identified 24 genomic loci significantly associated with module M1, of which 8 genomic loci were colocalized with eQTL hotspots (Figure 6b; Table S13, Supporting Information). To prioritize the candidate key regulators controlling the expression pattern of module M1 in GWAS loci, the promoter regions of lncRNAs in module M1 were extracted for enrichment analysis of binding motifs of transcription factors (TFs). Interestingly, we found that binding motifs of ERF3 in hot4 and ERF017 in hot14 were significantly enriched in promoters of lncRNAs in module M1 (Figure 6c). *ERF3* was reported to be involved in the regulation of seed weight in rice and wheat.^[^ ^15^ ^]^ Therefore, *ERF3* and *ERF017* may be the potential regulators of module M1.

The aforementioned findings suggest that the lncRNAs within module M1 may serve as candidate regulators of seed weight. To substantiate this hypothesis, *GmlncRNA10551*, a member of this module, was selected to elucidate the involvement of module M1 in seed weight modulation. *GmlncRNA10551* showed significant correlation with seed weight at both 14 and 21 DAF (Figures 2a and 6d). We obtained two independent transgenic overexpression lines (OE1*^10551^* and OE2*^10551^*), which exhibited significant expression increase of *GmlncRNA10551* relative to wild type (WT) in leaves (Figure 6e). Compared with WT, OE1*^10551^* and OE2*^10551^* exhibited a significant increase in 100‐seed weight, seed length, seed width, and seed thickness (Figure 6f,g; Figure S6a–d,f–h, Supporting Information). Meanwhile, there was no obvious change in seed number per plant and plant architecture between WT and transgenic lines (Figure S6e,i,j, Supporting Information). In addition to *GmlncRNA10551*, another TWAS‐significant lncRNA *GmlncRNA02765*, which exhibited a positive correlation with seed weight at both 14 and 21 DAF, was selected to validate the function of lncRNAs outside module M1 (Figures 2a and 6h). Two independent transgenic overexpression lines (OE1*^02765^* and OE2*^02765^*) were generated, both exhibiting significant upregulation of *GmlncRNA02765* compared to the WT in leaves (Figure 6i). Both transgenic overexpression lines (OE1*^02765^* and OE2*^02765^*) demonstrated a significant increase in 100‐seed weight, seed length, seed width, and seed thickness, without any alteration in seed number per plant or plant architecture (Figure 6j,k; Figures S6j and Figure S7, Supporting Information).

### 2.7 Target Genes Regulated by lncRNAs during Seed Development

**Figure 7:**
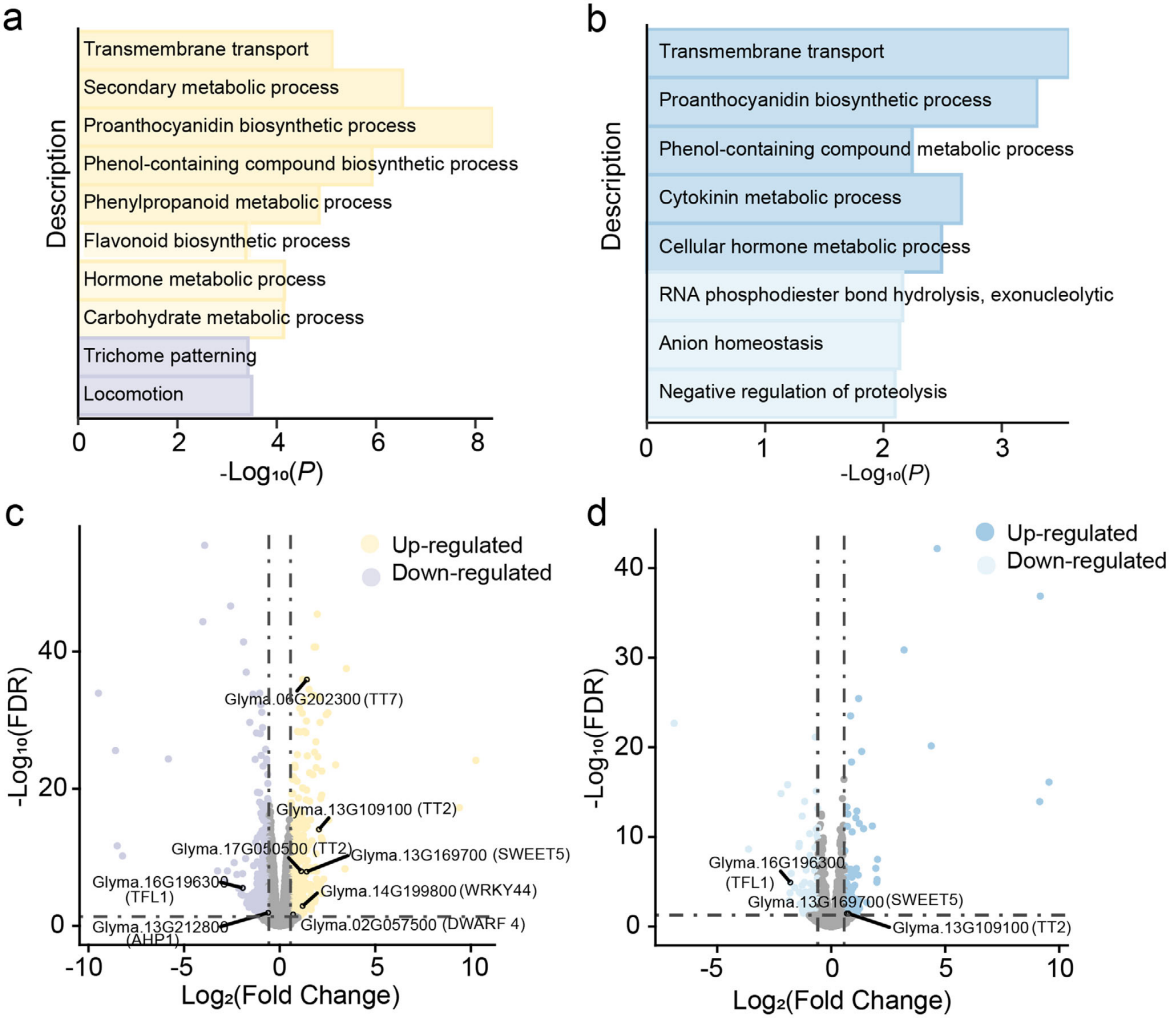
Analysis of DEGs between transgenic overexpression lines and WT. a) GO analysis of DEGs between OE1*^10551^* and WT. b) GO analysis of DEGs between OE1*^02765^* and WT. c) Volcano plot of DEGs between OE1*^10551^* and WT. The horizontal dashed line indicates the significance threshold (FDR = 0.05), while the vertical black dashed lines denote the fold‐change cutoff (|Fold Change| = 1.5). d) Volcano plot of DEGs between OE1*^02765^* and WT. The horizontal dashed line indicates the significance threshold (FDR = 0.05), while the vertical black dashed lines denote the fold‐change cutoff (|Fold Change| = 1.5).

To elucidate the molecular mechanisms by which *GmlncRNA10551* and *GmlncRNA02765* regulate seed weight, we performed a comparative transcriptomic analysis of seeds at 14 DAF between transgenic overexpression lines and WT. A total of 1056 differentially expressed genes (DEGs) were identified in OE1*^10551^* compared to WT, while 206 DEGs were detected in OE1*^02765^*. Among these, 123 DEGs showed overlapping expression patterns between the two transgenic lines. GO enrichment analysis showed that DEGs in OE1*^10551^* were significantly associated with biological processes such as hormone metabolic process and carbohydrate metabolism (**Figure** **7**a), while DEGs in OE1*^02765^* were significantly enriched in biological processes including transmembrane transport and cytokinin metabolic process (Figure 7b). Notably, several *Arabidopsis* homologs known to positively regulate seed size including *Glyma.06G202300* (*TT7*),^[^ ^16^ ^]^ *Glyma.13G109100*/*Glyma.17G050500* (*TT2*),^[^ ^16^ ^]^ *Glyma.14G199800* (*WRKY44*),^[^ ^17^ ^]^ and *Glyma.02G057500* (*DWARF4*) were up‐regulated in OE1*^10551^*,^[^ ^18^ ^]^while genes negatively associated with seed size *Glyma.16G196300* (*TFL1*) and *Glyma.13G212800* (*AHP1*) were down‐regulated (Figure 7c).^[^ ^19^ ^]^ In addition, *Glyma.16G196300* (*TFL1*), *Glyma.13G109100* (*TT2*), and *Glyma.13G169700* (*SWEET5*) were also identified in DEGs between OE1*^02765^* and WT (Figure 7d).^[^ ^20^ ^]^ These findings demonstrate that overexpression of *GmlncRNA10551* and *GmlncRNA02765* modulates key genes involved in flavonoid biosynthesis, hormone metabolism, sucrose transport, and photoperiodic regulation, ultimately enhancing seed weight.

## 3 Discussion

Seed weight is a crucial agronomic trait for enhancing soybean yield and quality. Although numerous genes associated with seed weight have been identified,^[^ ^21^ ^]^ the role of lncRNAs in regulating soybean seed weight has been scarcely reported. In this study, we employed a comprehensive approach integrating TWAS and eQTL analysis to identify TWAS‐significant lncRNAs and lncRNA‐lncRNA co‐expression module associated with seed weight. Additionally, we elucidated the impact of genetic variation on lncRNA expression. Through genetic transformation assays, we confirmed that two lncRNAs positively contribute to seed weight.

Over the past decade, advancements in transcript assembly algorithms and the reduction in sequencing costs have facilitated the identification of lncRNAs in various plants.^[^ ^2^ ^]^ In this study, we identified ≈17020 high‐confidence lncRNAs (Figure 1a). The RNA‐seq datasets used for lncRNA identification were generated using Illumina next‐generation sequencing (NGS) technology, which cannot completely avoid assembly errors in transcripts due to its inherent limitations in short read lengths. This makes it challenging to precisely identify the full‐length of lncRNAs and their isoforms. Recent research has highlighted that different isoforms of lncRNAs possess distinct biological functions.^[^ ^22^ ^]^ Third‐generation sequencing (TGS) technologies, primarily represented by PacBio or Oxford Nanopore, can capture full‐length transcripts, thereby mitigating transcript assembly errors and facilitating the identification of lncRNA isoforms.^[^ ^23^ ^]^ It is imperative to identify lncRNAs using TGS technologies in population transcriptome studies of soybean in the future. Additionally, the number of newly identified lncRNAs has not yet reached saturation, as lncRNAs generally exhibit low levels of expression conservation. Many lncRNAs (47%, 14 DAF; 49%, 21 DAF) were expressed in fewer than 20% of soybean accessions, and only 50 and 28 lncRNAs were expressed in all accessions at 14 DAF and 21 DAF, respectively (Figure 1c; Table S3, Supporting Information). These findings are consistent with research on lncRNAs in other plants.^[^ ^24^ ^]^ For instance, in maize, researchers analyzed two developmental stages of maize kernels (5 DAP with 228 accessions and 15 DAP with 368 accessions) and found that only 25 and 36 lncRNAs were expressed across all accessions, respectively.^[^ ^24a^ ^]^ Consequently, it is anticipated that employing a larger population will lead to the identification of more novel lncRNAs.

Precise regulation of gene expression is fundamental for tissue development in plants.^[^ ^25^ ^]^ In addition to PCGs, we observed a pronounced difference in both the number and expression levels of lncRNAs between 14 DAF and 21 DAF (Figure 1b,f). Across these two seed developmental stages, we identified 201 TWAS‐significant lncRNAs, of which only 24 were common to both stages (Figure 2b). These findings suggest that lncRNAs may play distinct roles at different stages of seed development. Previous studies have demonstrated that incorporating temporal expression data across multiple time points can enhance the identification of genetic variants with transient effects.^[^ ^10^, ^14^ ^]^ Therefore, integrating transcriptome data from various developmental time points during seed development will likely facilitate the identification of more spatiotemporally specific lncRNAs associated with seed weight. LncRNAs can regulate the expression of neighboring or distal PCGs and modulate chromatin structure by interacting with DNA, RNA, and proteins.^[^ ^26^ ^]^ Based on the correlation between expression levels of lncRNAs and adjacent PCGs,^[^ ^11^ ^]^ we predicted 149 lncRNA‐targeted PCGs across both seed development stages (Figure 2e; Table S5, Supporting Information). However, the reliability of these predictions has not been experimentally validated. There is an urgent need to develop new algorithms and experimental approaches to efficiently uncover the target genes of lncRNAs and elucidate molecular mechanisms underlying lncRNA regulation.

Emerging evidence implicates multiple ERF transcription factors in reciprocal regulatory networks with lncRNAs across plant species. In *Arabidopsis*, ERF84 physically associates with lncRNA *DANA2* to modulate drought stress adaptation,^[^ ^27^ ^]^ while in apple, light‐responsive anthocyanin biosynthesis involves *ERF109* activation by the upstream lncRNA *MdLNC499*.^[^ ^28^ ^]^ These examples illustrate a conserved, bidirectional interplay between ERFs and lncRNAs. However, the functional roles of soybean *ERF3* and *ERF017*, along with the mechanistic basis of their lncRNA regulation, remain unresolved. While our overexpression studies have validated the roles of *GmlncRNA10551* and *GmlncRNA02765* in regulating seed size by overexpression, developing robust large‐fragment knockout systems remains essential to further elucidate their precise molecular functions.

The local effects of trait‐associated variants on nearby gene expression have been extensively explored in plants and animals.^[^ ^29^ ^]^ However, the “omnigenic” model of complex traits posits that distal eQTLs collectively contribute substantially to trait variance by influencing broad gene regulatory circuits.^[^ ^30^ ^]^ Unlike cis‐regulatory loci, trans‐regulatory variants are dispersed throughout the genome, affecting gene or lncRNA expression directly or indirectly.^[^ ^31^ ^]^ In our study, major TWAS‐significant lncRNAs harbored multiple non‐redundant regulatory loci, highlighting the importance of both *cis* and *trans* effects in expression modulation (Figure 5a,b). Notably, accessions in the top 10% expression rank aggregated over 50% of superior eQTLs for each lncRNA (Figure 5b), as well as distal superior eQTLs for 100‐seed weight, which were concentrated in a small subset of accessions with exceptionally high seed weight (Figure 5c). These findings suggest that the effects of local and distal eQTLs on target lncRNA expression may act in opposite directions in most accessions, potentially due to stabilizing selection on expression or weaker selection pressures on numerous minor‐effect distal eQTLs during breeding. This aligns with hybrid studies showing that *cis*‐*trans* compensation is commonly observed within species such as *Arabidopsis*, mouse, and *Drosophila*,^[^ ^32^ ^]^ serving to stabilize overall gene expression.^[^ ^33^ ^]^ Overall, our results underscore the crucial role of distal eQTLs in influencing phenotypes by regulating lncRNA expression. Given their dispersion, minor effects, and opposing *cis*‐*trans* interactions, distal eQTLs are challenging to leverage in crop and animal breeding. Future soybean breeding should target both major‐effect proximal and minor‐effect trans‐regulatory loci to optimize networks and enhance control of agronomic traits.

## 4 Experimental Section

### Plant Materials and Growth Conditions

The soybean accessions were planted at the Liuhe experimental station of Jiangsu Academy of Agricultural Sciences in Nanjing in 2019. In alignment with the prior investigation wherein seeds were harvested at 21 DAF,^[^ ^9^ ^]^ seeds at 14 DAF were collected from 10:00 am to 11:00 am in August 2019 for total RNA extraction and 3′RNA‐seq library construction. The homozygous T3 transgenic plants and wild type were grown at the Baima experimental station of Nanjing Agricultural University in Nanjing in 2023 and 2024 for phenotype comparison.

### RNA Isolation, RT‐qPCR, and lncRNA Amplification

Total RNA was extracted from seeds at 14 DAF of natural accessions and leaves of transgenic lines using the RNAiso Plus kit (Takara, Beijing, China). First‐strand cDNA was synthesized using the PrimeScript RT reagent Kit with gDNA Eraser (Takara, Beijing, China) following the manufacturer's instructions. The transcripts of lncRNAs were amplified using cDNA as the template by Phanta Super‐Fidelity DNA Polymerase (Vazyme, Jiangsu, China).

The cDNA was used as the template for qPCR, which was performed using AceQ qPCR SYBR Green Master Mix (Vazyme, Jiangsu, China) on the CFX96 Touch Real‐Time PCR Detection System (Bio‐Rad, CA, USA) with three biological replicates. The relative expression levels of lncRNAs were normalized to the expression of *GmTubulin* gene (*Glyma.03G124400*) and calculated for each sample by the 2^−ΔCT^ method. The primers used in lncRNA amplification and RT‐qPCR are listed in Table S14 (Supporting Information).

### Identification of lncRNAs in the Soybean Genome

The raw reads from published strand‐specific RNA‐seq libraries were first filtered to remove adapter sequence and low‐quality bases by Fastp (version 0.21.0).^[^ ^34^ ^]^ Next, the clean reads were mapped to the soybean Williams 82 reference genome (Gmax_508_a4.v1.0) using Hisat2 (version 2.1.0) with the following parameters (for paired‐end reads, –rna‐strandness RF; for single‐end reads, –rna‐strandness R).^[^ ^35^ ^]^ Uniquely mapped reads were extracted by Samtools (Version 1.9).^[^ ^36^ ^]^ Subsequently, the uniquely mapped reads from each RNA‐seq dataset were used to assemble transcripts based on the Williams 82 reference genome annotation GTF file by StringTie (version 2.1.4) with the following command “stringtie –merge ‐c 1 ‐F 0.5 ‐T 0.5”.^[^ ^37^ ^]^ A stringent pipeline was implemented to identify lncRNA transcripts as previously described.^[^ ^38^ ^]^ First, all assembled transcripts from individual samples were merged into a GTF file using the StringTie –merge function. Next, the merged GTF file was compared to the reference genome annotation GTF file to confirm the positions and types of lncRNAs using Cuffcompare (Version 0.12.6).^[^ ^39^ ^]^ All transcripts shorter than 200 nt were discarded. Subsequently, transcripts with coding potential were discarded using CPC2 software.^[^ ^40^ ^]^ The remaining transcripts were aligned to the Pfam database (release 33.1; Pfam A) to exclude transcripts containing protein domains.^[^ ^41^ ^]^ To eliminate ribosomal RNAs, transfer RNAs, and small RNAs from consideration, the remaining transcripts were aligned against the Rfam database (version 14.6) utilizing the cmscan software.^[^ ^42^ ^]^ Finally, the filtered transcripts were considered as high confidently lncRNAs.

### Library Construction and Data Analysis of 3′ RNA‐seq

The 3′ RNA‐seq libraries were constructed using total RNA from seeds at 14 DAF for 238 soybean accessions as described.^[^ ^9^ ^]^ Briefly, after ≈1 µg RNA was fragmented, oligo‐dT index primers were used to synthesize the first‐strand cDNA. After synthesis of double‐stranded DNA, end repair, dA‐tailing, adapter ligation, and PCR amplification were performed to generate 3′ RNA‐seq libraries. The libraries were sequenced on NovaSeq platform (Illumina) for 150 bp paired‐end reads.

Raw data were filtered to remove adapter sequences and low‐quality bases by Fastp (version 0.21.0).^[^ ^34^ ^]^ The clean data were mapped to the soybean reference genome (Gmax_508_a4.v1.0) using Hisat2 (version 2.1.0).^[^ ^35^ ^]^ Only uniquely mapped reads were extracted for downstream analysis. The gene and lncRNA count matrices were generated using featureCounts (v2.0.3), and the expression level (transcripts per million, TPM) was calculated for each gene and lncRNA by edgeR.^[^ ^43^ ^]^

### TWAS Analysis

The phenotypic data for 100‐seed weight of 238 accessions were obtained from the previous research.^[^ ^9^ ^]^ After discarding lncRNAs with low expression levels (TPM < 0.5 in more than 95% of accessions), the Pearson correlation coefficients (PCCs) were calculated between the expression levels of lncRNAs and 100‐seed weight phenotypes among the 238 soybean accessions. *P* values were corrected using the Benjamini‐Hochberg correction method. The lncRNAs with FDR < 0.05 were considered as TWAS‐significant lncRNAs.

### Target Gene Prediction for lncRNAs

Based on previous research in rice,^[^ ^11^ ^]^ PCCs between lncRNA and adjunct PCGs in 50 kb flanking regions were calculated for each lncRNA. The PCGs with *P* value < 0.01 were considered as target genes of the corresponding lncRNA.

### Plasmid Construction and Transformation

The sequences of candidate lncRNAs were amplified using seed cDNA and ligated into the *BamHI/XhoI* sites of the pFGC5941 vector containing a 35S promoter by the ClonExpress II One Step Cloning Kit (Vazyme, Jiangsu, China). The recombinant vectors were introduced into *Agrobacterium tumefaciens* strain EHA101 and used to transform soybean cultivar Williams 82 by *A. tumefaciens*‐mediated transformation. Due to soybean's inherent transformation inefficiency, 6 and 8 independent T0 transgenic lines were obtained for overexpression of *GmlncRNA10551* and *GmlncRNA02765*, respectively. However, extensive chimerism in primary transformants permitted recovery of just two stably inherited overexpression lines per lncRNA. The expression levels of lncRNAs in wild‐type and transgenic plants were tested by RT‐qPCR analysis.

### Genetic Diversity Analysis

The SNP data of 2898 soybean accessions were downloaded from the Genome Variation Map (GVM) database in BIG Data Center (GVM000063). To transfer coordinates of the SNPs from the soybean reference genome of ZH13 to William82, genome alignment was performed by Minimap2 (version 2.24‐r1122) with parameters (‐cx asm5 –cs) and created chain file by transanno (version 0.3.0; https://github.com/informationsea/transanno). Then, the coordinates of the SNPs were updated by CrossMap (version 0.6.4).^[^ ^44^ ^]^ Using 100 kb window and 10 kb step, π value and *F*st value between landraces and wild soybeans were calculated via VCFtools (version 0.1.16). The selective sweeps were defined as regions with the top 5% of *F*st and reduction of diversity (ROD) values among genomic regions.^[^ ^45^ ^]^

### Identification of eQTLs and Hotspots of lncRNAs

The genotype and lncRNA expression of 238 soybean accessions were used for eQTL detection. SNP datasets of soybean accessions were obtained from the previous study.^[^ ^9^ ^]^ After discarding lncRNAs with low expression level (TPM < 0.5 in more than 95% of accessions), the expression of each lncRNA was quantile normalized and used as a phenotype for GWAS. Using LOCO function in GCTA software, eQTL‐lncRNA associations were identified according to a threshold *P* < 1e^−5^.^[^ ^46^ ^]^ All eQTL‐lncRNA associations were clumped using LD‐threshold (*r* ^2^) of 0.2 in a 250 kb radius by PLINK. The lead SNP (*P* < 3.6 e^−7^; 1/n, n represents the total number of SNPs used in eQTL detection) with more than two LD proxies were retained as putative eQTL.^[^ ^47^ ^]^ The putative eQTLs, which were identified in a LD block via PLINK (–blocks no‐pheno‐req no‐small‐max‐span –blocks‐max‐kb 5000 –blocks‐strong‐lowci 0.70 –blocks‐strong‐highci 0.98 –blocks‐recomb‐highci 0.9 –blocks‐inform‐frac 0.8), were further merged and represented by the most significant lead SNP.^[^ ^47^ ^]^ According to the distance (cutoff: 1Mb) between lead SNP and associated lncRNAs, the eQTLs were classified as local or distal eQTLs in subsequent analysis. Using 1 Mb sliding window with 100 kb step, distal eQTL hotspots were detected based on the mapped lncRNA numbers greater than the threshold (16 for 14 DAF and 15 for 21 DAF), which was calculated from the distribution of the maximum number of mapped lncRNAs in 1000 random permutations with shuffling positions of distal eQTLs.

### Construction of lncRNA co‐Expression Module

The lncRNA co‐expression modules were constructed using WGCNA software in R with parameters (networkType = “unsigned”, corType = “pearson”, deepSplit = 2, minModuleSize = 30, and mergeCutHeight = 0.25).^[^ ^48^ ^]^ Pearson correlations between the PCA values of the lncRNA co‐expression modules and the 100‐seed weight were calculated among 238 soybean accessions. The significant correlation between lncRNA modules and seed weight was determined using a *P* value threshold of less than 0.05.

### Identification of Distal eQTLs for Co‐Expression Modules

Using module expression as a phenotype, GWAS was conducted using the mixed linear model of GCTA (version 1.92.3) with the parameters (–make‐grm –pca). The significance level (*P* value < 1.85 e^−7^) was calculated using the GEC software.^[^ ^49^ ^]^ To identify the candidate transcription factors in the distal eQTL of the module, promoter sequences (2 kb) of all lncRNAs in the module were extracted for motif enrichment analysis by SEA function in MEME Suite.^[^ ^50^ ^]^

### Analysis of Differentially Expressed Genes

Seeds were collected from both transgenic lines and wild type at the 14 DAF with three biological replicates. RNA‐seq libraries were constructed using the VAHTS Universal V8 RNA‐seq Library Prep Kit for Illumina (Vazyme, Jiangsu, China), following the manufacturer's protocol. Sequencing was performed on the Novaseq platform (Illumina) with paired‐end 150 bp reads. Raw RNA‐seq data were processed using Fastp (v0.21.0) for quality control, followed by alignment to the soybean reference genome (Gmax_508_a4.v1.0) using Hisat2 (v2.1.0).^[^ ^34^, ^35^ ^]^ Gene counts were quantified with featureCounts (v2.0.3), and differential expression analysis was conducted using the R package edgeR.^[^ ^43^ ^]^ DEGs were identified with a threshold of FDR < 0.05 and |Fold Change| > 1.5.

## Conflict of Interest

The authors declare no conflict of interest.

## Author Contributions

X.W. and X.J. contributed equally to this work. Q.S. conceived and designed the research. X.W., X.Y., Z.Z., Z.Z., and W.Y. performed the experiments. X.W., X.J., L.W., and W.J. analyzed the data. X.W., X.J., and Q.S. wrote the manuscript. All authors read and approved the final manuscript.

## Supporting information



Supporting Information

Supporting Information

## Data Availability

The data that support the findings of this study are openly available in Sequencing data of RNA‐seq are available at National Genomics Data Center at https://www.cncb.ac.cn, reference number PRJCA031929 and PRJCA040146.
