# Supplementary material for: Transcriptome‐Wide Association Uncovers LncRNAs Controlling Seed Weight in Soybean
Source: Adv Sci (Weinh). 2025 Aug 11;12(41):e16794. doi: 10.1002/advs.202416794 (PMC12591141; doi:10.1002/advs.202416794)
Supplement: Supplementary file 1 — Supporting Information [file ADVS-12-e16794-s002.pdf]

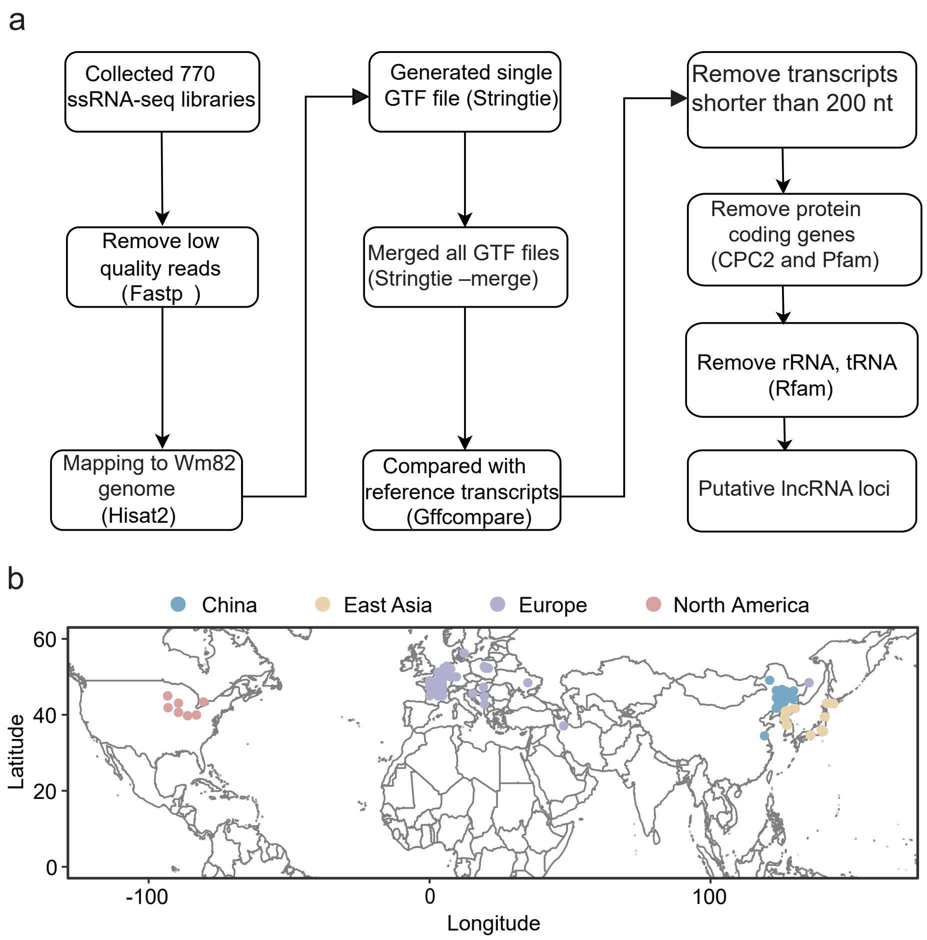

Figure S1. Identification of soybean lncRNA and geographical distribution of 238 soybean accessions. a) Pipeline for lncRNA identification. b) Geographical distribution of 238 soybean accessions. The color of the dots represents different geographical locations corresponding to the 238 accessions.

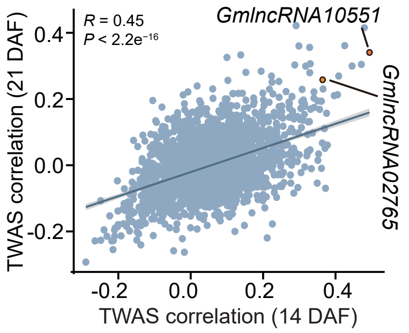

Figure S2. The distribution of Pearson correlation coefficients (PCCs) of TWAS at 14 DAF and 21 DAF. The y-axis represents the PCCs between lncRNA expression and 100-seed weight at 21 DAF. The x-axis represents the PCCs between lncRNA expression and 100-seed weight at 14 DAF.

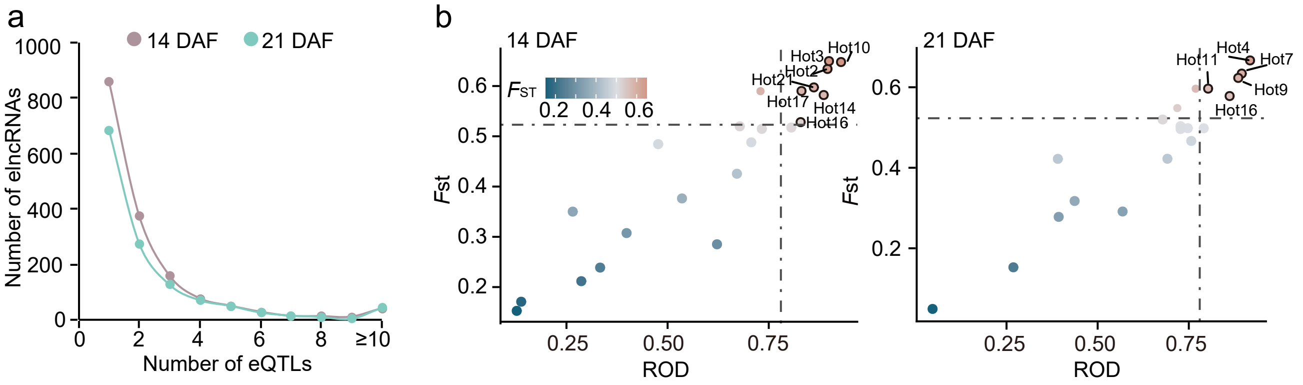

Figure S3. The numbers of eQTLs corresponding to the elncRNAs and identification of domesticated eQTL hotspots. a) Counts of elncRNAs regulated by different numbers of eQTLs. b) The eQTL hotspots under artificial selection. The dashed lines indicate the significant genome-wide threshold (top 5%) of selection signals (*F*st, 0.52; ROD, 0.78; wild vs. landrace).

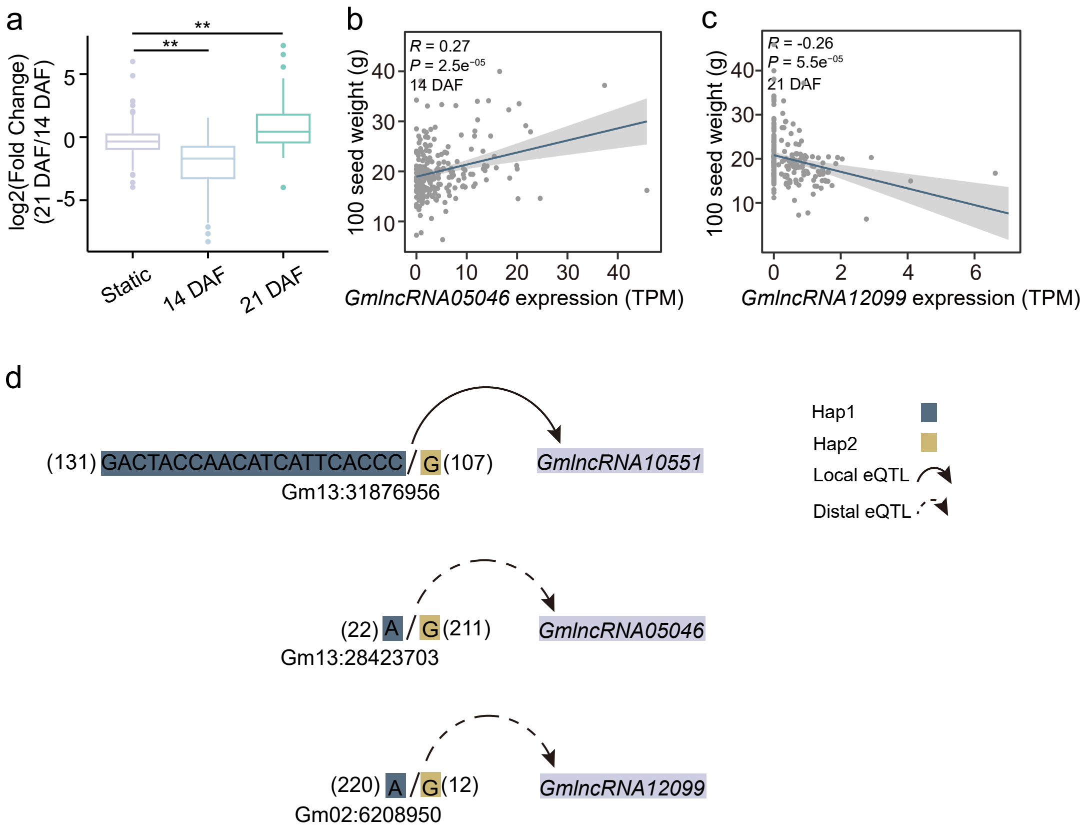

Figure S4. Static and dynamic eQTLs regulate lncRNAs expression. a) Expression changes of static and dynamic elncRNAs between 14 and 21 DAF. Two-sided Wilcoxon rank sum test was used for statistical analysis, $^{**}P < 0.01$. b) Scatter plot of correlation between *GmlncRNA05046* expression and 100-seed weight. c) Scatter plot of correlation between *GmlncRNA12099* expression and 100-seed weight. d) Spectrum of two haplotypes (Hap1 and Hap2) of eQTLs associated with TWAS-significant lncRNAs in 238 soybean accessions. Numbers in parentheses indicate the count of accessions carrying each haplotype. *GmlncRNA10551* regulated by a static eQTL. *GmlncRNA05046* regulated by a dynamic eQTL at 14 DAF. *GmlncRNA12099* regulated by a dynamic eQTL at 21 DAF.

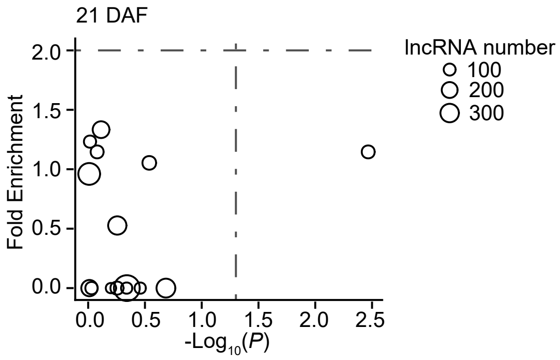

Figure S5. Identification of the key co-expression module regulating 100-seed weight at 21 DAF. The y-axis represents the fold enrichment of TWAS-significant lncRNAs in each module. The x-axis represents the P value of the Pearson correlation between modules and 100-seed weight. The horizontal and vertical dashed lines represent the corresponding threshold (Fold Enrichment ≥ 2; $P < 0.05$).

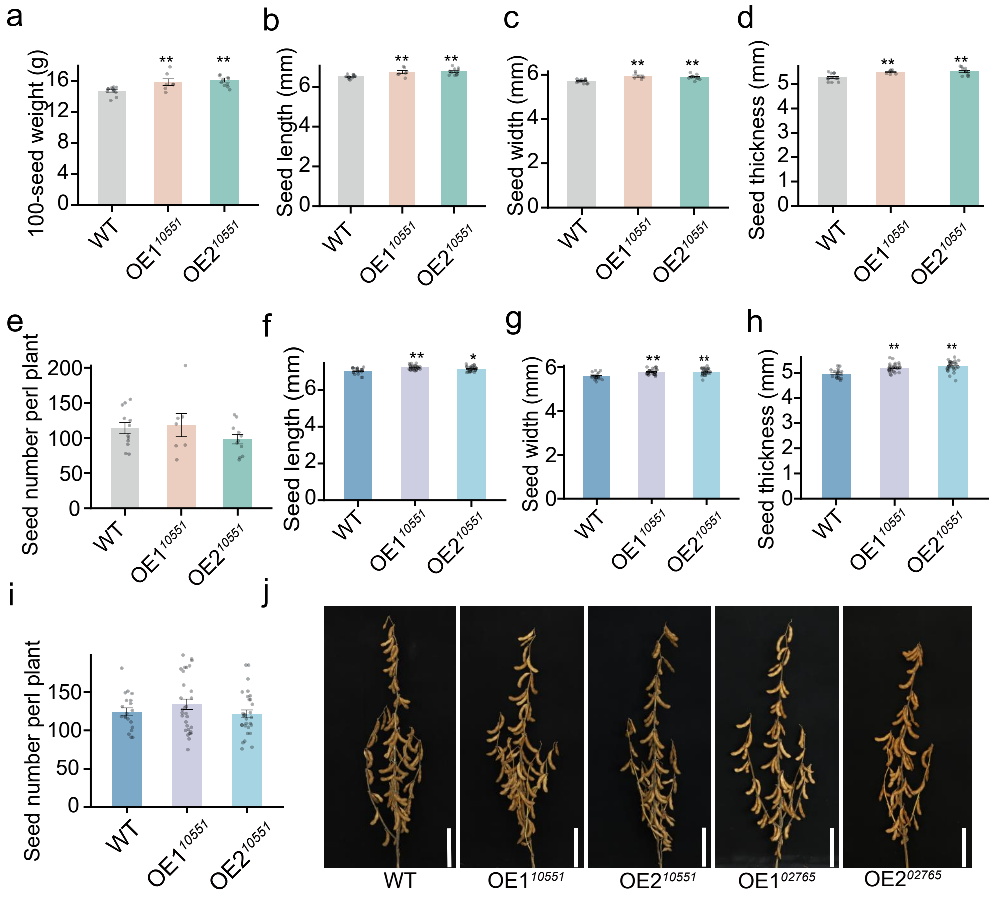

Figure S6. The phenotype changes of transgenic lines of *GmlncRNA10551* compared to WT. a-e) 100-seed weight a), seed length b), seed width c), seed thickness d) and seed number per plant e) of transgenic lines overexpressing *GmlncRNA10551* and WT in 2023 (n = 12, 7, 11 biological independent plants for WT, OE1 and OE2, respectively). Statistical analysis was performed using a two-tailed *t*-test. Data are presented as means ± SEM. $^{**}P < 0.01$ and $^{*}P < 0.05$. f-i) Seed length f), seed width g), seed thickness h) and seed number per plant i) of transgenic lines overexpressing *GmlncRNA10551* and WT in 2024 (n = 20, 30, 30 biological independent plants for WT, OE1 and OE2, respectively). Statistical analysis was performed using a two-tailed *t*-test. Data are presented as means ± SEM. $^{**}P < 0.01$ and $^{*}P < 0.05$. j) Plant architecture of transgenic lines and WT lines at the harvest stage. Scale bar: 10 cm.

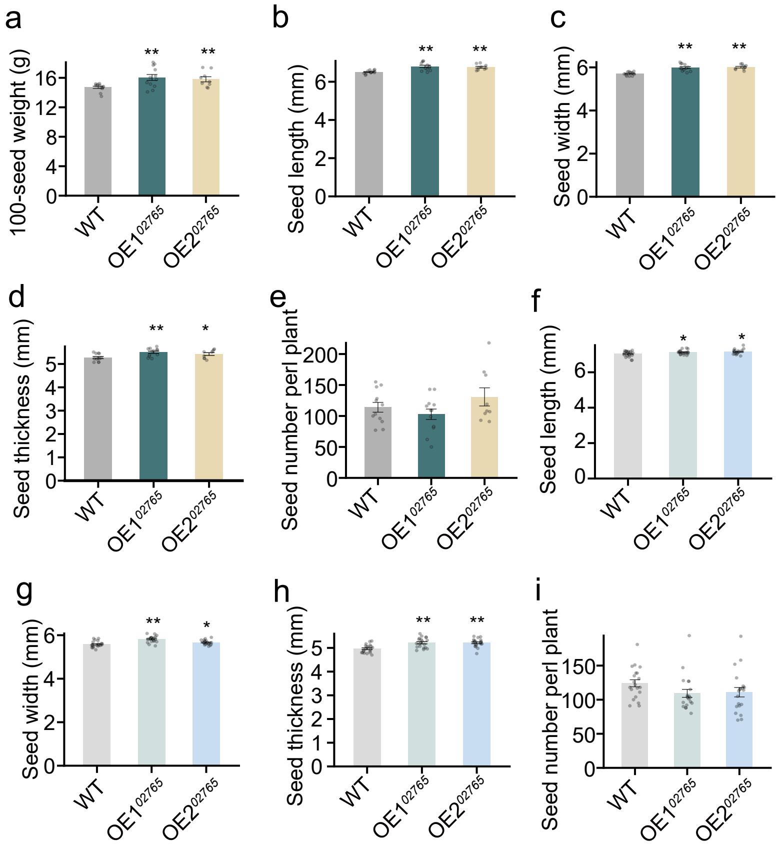

Figure S7. The phenotype changes of transgenic lines of *GmlncRNA02765* compared to WT. a-e) 100-seed weight a), seed length b), seed width c), seed thickness d) and seed number per plant e) of transgenic lines overexpressing *GmlncRNA02765* and WT in 2023 (n =12, 12, 9 biological independent plants for WT, OE1 and OE2, respectively). Statistical analysis was performed using a two-tailed *t*-test. Data are presented as means ± SEM. $^{**}P < 0.01$ and $^{*}P < 0.05$. f-i) Seed length f), seed width g), seed thickness h) and seed number per plant i) of transgenic lines overexpressing *GmlncRNA02765* and WT in 2024 (n = 20 biological independent plants for WT, OE1 and OE2, respectively). Statistical analysis was performed using a two-tailed *t*-test. Data are presented as means ± SEM. $^{**}P < 0.01$ and $^{*}P < 0.05$.
